# Orientation Dependent Mechanical Responses and Plastic Deformation Mechanisms of ZnSe Nano Films under Nanoindentation

**DOI:** 10.3390/nano11113014

**Published:** 2021-11-10

**Authors:** Chao Xu, Futi Liu, Chunmei Liu, Pei Wang, Huaping Liu

**Affiliations:** 1 Faculty of Science, Yibin University, Yibin 644007, China; chaoxu@wust.edu.cn; 2 Key Laboratory of Computational Physics, Yibin University, Yibin 644007, China; 3 College of Science, Wuhan University of Science and Technology, Wuhan 430081, China; 4 Academy for Advanced Interdisciplinary Studies, Department of Physics, Southern University of Science and Technology (SUSTech), Shenzhen 518055, China; pwsustech@outlook.com; 5 School of Aerospace Engineering, Huazhong University of Science and Technology, Wuhan 430074, China; huaping_liu@163.com

**Keywords:** indentation, plastic deformation, ZnSe nano film, molecular dynamics

## Abstract

Although ZnSe has been widely studied due to its attractive electronic and optoelectronic properties, limited data on its plastic deformations are available. Through molecular dynamics simulations, we have investigated the indentations on the (001), (110), and (111) planes of ZnSe nano films. Our results indicate that the elastic modulus, incipient plasticity, elastic recovery ratio, and the structural evolutions during the indenting process of ZnSe nano films show obvious anisotropy. To analyze the correlation of structural evolution and mechanical responses, the atomic displacement vectors, atomic arrangements, and the dislocations of the indented samples are analyzed. Our simulations revealed that the plastic deformations of the indented ZnSe nano films are dominated by the nucleation and propagation of 1/2<110> type dislocations, and the symmetrically distributed prismatic loops emitted during the indenting process are closely related with the mechanical properties. By studying the evolutions of microstructures, the formation process of the dislocations, as well as the formation mechanisms of the emitted prismatic loops under the indented crystalline planes are discussed. The results presented in this work not only provide an answer for the questions about indentation responses of ZnSe nano films, but also offer insight into its plastic deformation mechanisms.

## 1. Introduction

As an important II–VI semiconductor, zinc selenide (ZnSe) has attracted great attention because of its outstanding electronic and optoelectronic properties and potential applications [1,2,3,4,5,6,7]. ZnSe exhibits several favorable characteristics, including a direct bandgap, low resistivity, chemical stability, and emission/absorption signals ranging from the ultraviolet to the infrared. Because of these excellent properties, ZnSe materials are widely used in nonlinear optical devices, flat panel displays, light emitting diodes (LEDs), lasers, logic gates, transistors, etc. [8,9,10,11,12,13,14]. In addition to thin films and macroscopic crystals, ZnSe has been obtained in a great variety of morphologies in the nanometric range, that make it suitable for applications such as field emitters, sensors, actuators, and many other optoelectronic devices [9,11,15,16].

In spite of these applications, ZnSe materials present mechanical properties much weaker than those of other commonly used semiconductors and have hardness as low as those of soft metals [17,18]. Due to defect generation and propagation during operation, the lifetime of ZnSe based devices is still limited [18]. Therefore, in order to improve the reliability of ZnSe based devices, the mechanical properties of ZnSe need to be considered when designing of ZnSe based devices [19]. With the development of nanotechnology, the nanoindentation technique provides a powerful routine to investigate the deformation behaviors and measure the mechanical properties of materials at nanometer scale.

Experimentally, Grillo et al. [18,20] studied the nanoindentation behaviors of ZnSe single crystal on (001) plane and Zn_1__−x_Be_x_Se heteroepitaxial layers. They found that the orientation of the Berkovich indenter significantly affect the nanoindentation response. Combined with the nanoindentation and cross-sectional transmission electron microscopy techniques, Jian and Lin [21] investigated the Berkovich nanoindentation-induced pop-in effects, the mechanical deformation mechanisms, and the formed dislocation loops in ZnSe thin films. Jian and Tseng [22] also reported the nanomechanical properties and the contact-induced deformation behaviors of ZnSe thin film with the same techniques. Yau et al. [23] employed cathodoluminescence (CL) to reveal the dynamics of recombination-enhanced dislocation of ZnSe materials under nanoindentation. With these methods, Wen et al. [24] further examined the effects of the nanoindentation-induced residual stress of single-crystalline ZnSe. They not only evaluated the dislocation mobility by the nanoindentation technique, but also visualized the resultant dislocation and microcracks using CL spectroscopy and mapping to compare the nanoindentation-induced residual stresses of the various ZnSe samples.

Although these experimental attempts have contributed to the understanding of the deformation mechanisms and mechanical properties of ZnSe, some questions remain challenging owing to the limit in technical difficulty and cost. For example, full observation of the microstructure evolution, as well as the formation and propagation process of dislocations at the atomic level, is still challenging. Fortunately, molecular dynamics (MD) simulation of nanoindentation can make up for the shortcomings of experimental technology. MD simulations have been widely used to investigate the nanoindentations of materials [25,26,27,28,29,30,31,32]. It can achieve the mechanical properties of materials. Particularly, it can easily trace the microstructure evolutions of materials at nanoscale, including dislocations and transformations during the deformation processes.

In this work, our purpose is to study the mechanical responses and plastic deformations of ZnSe nano films under indentations, and carefully document the mechanisms and sequence of physical process occurring underneath the indentations through molecular dynamic simulations. The results of this work are expected to help to a better understanding of the basic deformation behaviors of ZnSe nano films under indentations, and would provide reference data to improve the design and ultimately performance of the related ZnSe based devices.

## 2. Methods

**Figure 1:**
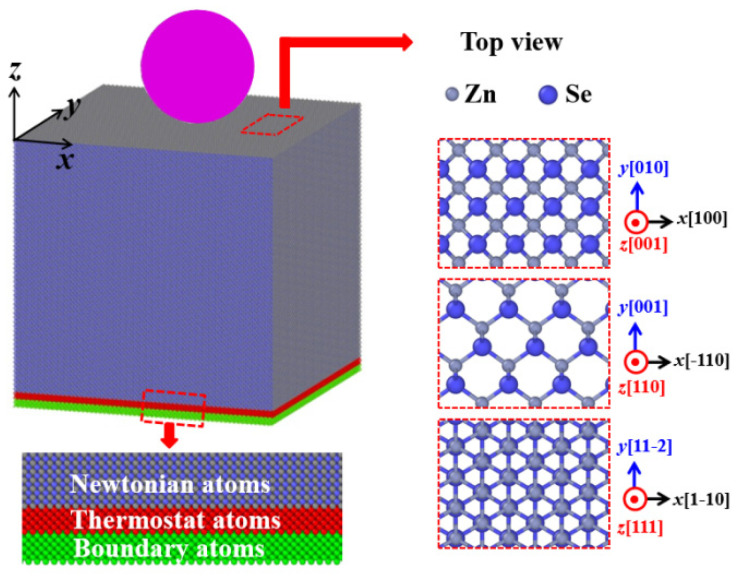
Schematic of indentation simulation setup and the crystal cell along the selected crystal directions.

To explore the effects of crystal orientations on the mechanical properties of zinc blende ZnSe nano films, three models with surface orientations on (001), (110) and (111) are prepared, respectively, for nanoindentations. The detail dimensions and atoms for all the constructed models are listed in Table 1. The schematic of indentation simulation setup as well as the crystal cell along the selected crystal directions are shown in Figure 1. Before the indentation simulations, the constructed ZnSe nano films were relaxed to obtain their equilibrium structures. During the relaxation process, periodic boundary conditions were imposed along x, y and z directions. The models were first optimized through conjugate gradient algorithm to achieve minimum equilibrium energy, and then further equilibrated at 0 bar and 1 K for 200 ps using an NPT ensemble. After this procedure, the equilibrated ZnSe models were used for the indenting simulations. During the indenting simulations, free boundary conditions were imposed in z direction, while periodic boundary conditions were imposed in x and y directions. In the simulated ZnSe nano films, atoms were divided into three types, namely, fixed atoms, thermostatic atoms, and Newtonian atoms, as illustrated in Figure 1. The lowest seven layers of atoms in the model were fixed at their initial lattice position to provide structural stability and prevent the sample from moving during the simulation process. The adjacent seven layers of atoms were set as the thermostatic atoms to ensure reasonable outward heat conduction, and the rest of atoms were set as the Newtonian atoms to obey classic Newton’s second law. A spherical rigid indenter with a diameter of 12.0 nm was initially positioned above the top surface of the ZnSe substrate. To describe the interaction between the indenter and the ZnSe substrate, Kelchner’s theoretical model was employed, which has been widely used in the nanoindentation simulations of different material systems [27,28,29,31,32,33,34]. Kelchner’s theoretical model introduces a repulsive potential to simulate the frictionless contact between the indenter and the indented materials [35]. In our simulations, each atom in the ZnSe nano films interacts with the idealized spherical rigid indenter to experience a force F(r) = −K(r-R)^2^, where K is the force constant, R represents the radius of the spherical rigid indenter, and r refers to the distance from the atom of the ZnSe model to the center of the indenter. When r is larger than R, no interaction is considered between the indenter and the indented samples.

Molecular dynamics simulations were performed by using the open source code LAMMPS [36]. Interactions among the atoms in the indented ZnSe nano films were depicted through the Stillinger–Weber potential model developed by Zhou et al. [37]. This interatomic potential is capable of predicting the crystal structures and defects for semiconductor compounds composed of the major II-VI elements Zn, Cd, Hg, S, Se, and Te. The reliability of this potential is widely tested by accurately reproducing the experimental cohesive energies, lattice constants, bulk moduli of all binary compounds, and capturing the transformation between elemental, solid solution, and compound phases. Most importantly, two successful growth simulations verify the predictive power on structure evolution and defects configurations such as misfit dislocations, stacking faults, and subgrain nucleation [37]. During the simulations of the indenting process, the indenter was initially positioned 1.0 nm above the top surface. Then, it was set moving down at a constant velocity of 0.01 nm/ps. In our simulations, the indenter was first controlled to penetrate into the sample substrate gradually to a maximum indentation depth of 5.0 nm. In addition, the indenter was then retracted at the same velocity to its initial position. All structural analysis and visualization of atomic configurations were performed by using the open-source program OVITO [38]. Identify diamond structure (IDS) method was adopted to analyze the structure evolution and defect nucleation, which can identify and visualize the stacking faults and dislocations [39]. The dislocation extraction algorithm (DXA) was also used to identify the defects of the indented models, which can analyze the dislocations, determine the Burgers vectors, and output dislocation lines [40,41].

## 3. Results and Discussions

### 3.1. Testing of Interatomic Potential

The selection of interatomic potential directly determines the reliability and accuracy of the results calculated by molecular dynamics simulations. Therefore, before the simulations of indentations, we systematically tested the interatomic potential by reproducing the structural, elastic, and other important properties of ZnSe. The calculated structural parameters, as well as the elastic properties, are listed in Table 2. The data from previously reported experimental and theoretical work are also listed for comparison [42,43,44,45]. As shown in Table 2, the computed equilibrium structural parameter of ZnSe in our work is in reasonable agreement with the available data from experiments and first principle calculations. In addition, the elastic constants (namely C_11_, C_12_, and C_44_), bulk modulus, shear modulus, Youngs modulus, as well as Poisson’s ratio were also obtained. It can be seen that our results are basically consistent with the reported results, indicating that the interatomic potential selected in our simulations can reasonably reproduce the elastic properties of ZnSe.

**Figure 2:**
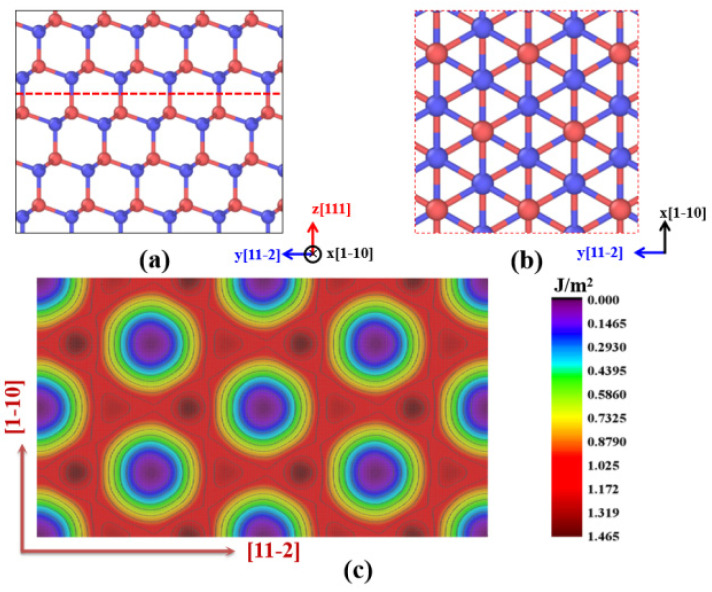
The constructed ZnSe nano film model and the results of the calculated generalized stacking fault energy: (**a**) Front view of the atomic arrangement of the model; (**b**) the atomic arrangement of the model; (**c**) generalized stacking fault energy surface of (111) plane.

Studying the indenting behaviors of ZnSe nano films will inevitably involve the plastic deformation of the indented ZnSe crystal. Whether the interatomic potential selected in the calculation can accurately describe the plastic deformation of ZnSe is of particular importance. Therefore, to evaluate the selected interatomic potential in our simulations, the generalized stacking fault energy surface (γ surface) has been calculated. It can derive dislocation core properties, such as core width, and the energy barrier for dislocation motion on a specific slip plane. The definition of generalized stacking fault energy (W_stf_) can be described by the following formula: W_stf_ = (E_f_ − E_0_)/A, where E_f_ and E_0_ refer to the potential energy of ZnSe crystal with and without stacking fault, and A represents the stacking-fault area. According to the reported results, the primary slip system for sphalerite ZnSe is (111)<110> [46,47]. Thus, in our calculations, we constructed a model of ZnSe crystal with three dimensions along [1-10], [11-2] and [111] directions. The front view and top view of the atomic arrangement can be found in Figure 2a,b.

In our calculations, the constructed ZnSe models are divided into two parts by the (111) crystalline plane, as illustrated as the dash line in Figure 2a. By rigidly displacing two halves of the ZnSe crystal on the (111) crystalline plane along [1-10] and [11-2] directions, the stacking fault can be generated. Through optimizing the obtained structure by the CG algorithm, the potential energy of the ZnSe system can be calculated. As the displacement proceeds, the potential energy of the ZnSe crystal changes as the atoms slip by each other, and we can get the generalized stacking fault energy, as well as the γ surface (generalized stacking fault energy surface), as shown in Figure 2c. According to the calculated γ surface, we can see that the shortest glide magnitude for the dislocations is described by the Burgers vector of b = 1/2<1-10>. In addition, three nonequivalent saddle points located at 1/6<11-2>a_0_, 1/3<11-2>a_0_, and 1/4<1-10>a_0_ on the γ surface can determine the energy barriers to slip along the <11-2> or <1-10> directions. For sphalerite structure, all-important energy configurations in the generalized stacking fault energy landscape are along the [11-2] and [1-10] directions. Therefore, we have paid particular attention to the generalized stacking fault energy along these two directions, and the related generalized stacking fault energy curves are shown in Figure S1 in the supplemental materials. In our calculations, we find that the generalized stacking fault energy curve of <11-2>{111} system is asymmetric, while that of the <1-10>{111} system is symmetric. The maximum stacking fault energy of the <11-2>{111} system is about 1.4 J/m^2^, which agrees well with the first principles calculation result (1.1 J/m^2^) [48]. In addition, the energy barrier along the [11-2] direction is higher than that in the [1-10] direction, indicating that it is energetically more favorable to glide along [1-10] direction. This result is in good agreement with previous experimental results [48], indicating the reliability of our calculations.

### 3.2. Mechanical Responses of ZnSe Nano Films under Indentations

**Figure 3:**
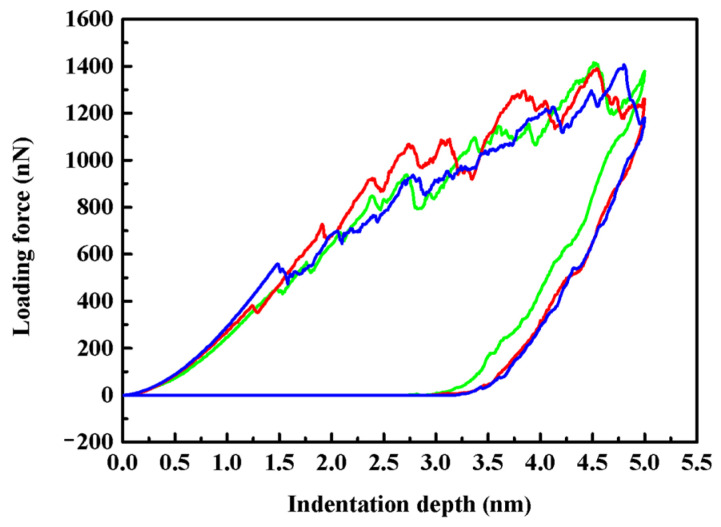
Loading forces as a function of indentation depth (P−h curves) for (001), (110), and (111) planes. Green, red, and blue lines represent the data of (001), (110), and (111) planes, respectively.

In our simulations, (001), (110) and (111) crystalline planes of ZnSe nano films were indented, and the corresponding loading forces versus indentation depth (P–h) curves for full cycles of indenting and retracting process at 1 K are illustrated in Figure 3. Obvious anisotropies on the indenting responses of the studied crystalline planes are found when comparing the curves corresponding to the loading and unloading process of the indenter. During the initial indenting process, the elastic deformation of the indented crystalline planes occurs, and the loading forces for all the indented crystalline planes increase monotonically with the indentation depth. As can be seen in Figure 3, when the indenter penetrates into these crystalline planes and produces the same indentation depth, the required loading forces on these planes are different. The loading force for the (111) plane is the greatest, followed by (110) plane, while that required for (001) plane is the lowest. As is known, when a greater loading force is required for a certain indentation depth during the elastic deformation stage of the indenting process, it indicates that it is more difficult to deform, resulting from the greater of elastic modulus. Therefore, anisotropy exists in the mechanical properties of the indented crystalline planes. We can speculate that the elastic modulus of the (111) plane is the highest, followed by (110) plane, while that required for (001) plane is the lowest.

As illustrated in Figure 3, when the depth that the indenter penetrated into the crystalline planes exceeding a critical value, an obvious drop of the loading forces can be observed in all the P-h curves. This phenomenon is the so-called pop-in event, and produces discontinuous steps in the load-displacement curves [27,29,31]. Such pop-in event was reported to correspond to unambiguous transitions from reversible elastic to irreversible plastic deformations during the earliest stages of the indenting process, and has been agreed to be a phenomenon correlated with the nucleation of dislocations [29,31]. As shown in the figure, the three indented planes reveal different extension of the elastic regime. The load drop associated with the initiation of plasticity occurs at different depths of 1.48 nm, 1.25 nm and 1.49 nm, respectively, for the indented (001), (110) and (111) planes, and the corresponding critical loading force are 457.3 nN, 379.1 nN and 557.7 nN. Therefore, we can speculate that plastic deformations occur first on (110) plane, followed by (001) and (111) plane. When the indenter was continually penetrated deeper into the samples, several significant load drop events happened, accompanied with the fluctuation of the loading forces, indicating more plastic events.

**Figure 4:**
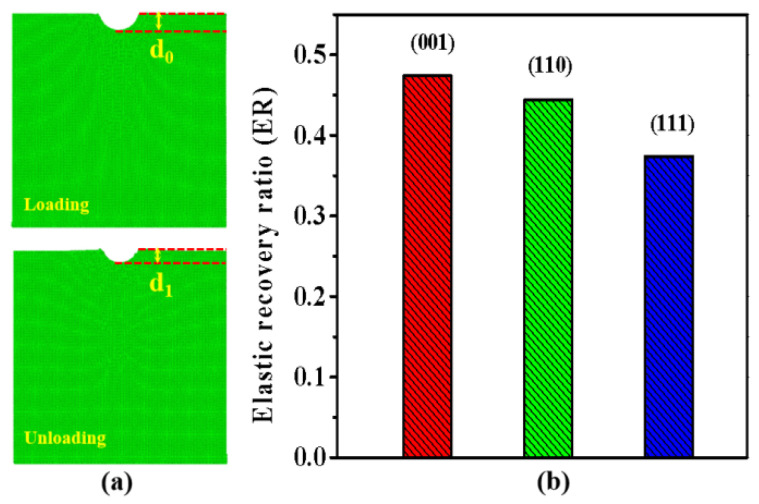
(**a**) Parameters for the definition of elastic recovery ratio after the retraction of indenter; (**b**) The elastic recovery ratio of the indented (001), (110) and (111) crystalline planes.

After the indenter retracted to its initial position, the morphologies of indentations left in the substrates were checked. As shown in Figure 4a, the final indentations were not maintained with the depth of 5 nm. Instead, all of them recovered to a certain extent. It is known that, when the indenter is pressed into the ZnSe sample, it exerts loadings on the sample, and compress the region under the indenter by pushing the surface of the indented surface downward. In comparison, when the indenter is retracted, the corresponding loadings would gradually vanish, and the compressed region under the indentations would have a chance to recovery in some extent. To quantitatively evaluate the elastic recovery of the indented planes for ZnSe, we have introduced a quantity named elastic recovery ratio (ER), whose definition can be described by the formula ER = (d_0_ − d_1_)/d_0_. In this formula, d_0_ and d_l_ represent the maximum depth of the indentation during the loading process and the final depth of the indentation after the indenter was retracted. The value of d_1_ is estimated by taking the depth when the loading force was reduced to zero during the unloading process. As shown in Figure 4b, we illustrate the elastic recovery ratio of the indented planes for ZnSe. As can be seen in the figure, the elastic recovery ratios on the indented crystalline planes of ZnSe nano films show obvious anisotropy. Among them, the recovery ratio of (001) plane is the highest (47%), followed by (110) plane (44%), and that of the (111) plane (37%) is the lowest. For comparison, we also calculated the elastic recovery ratio of zinc-blende AlN and GaN according to the reported data [49,50]. For zinc-blende AlN and GaN, the estimated elastic recovery ratios of different indented planes are in the range of 35–40% and 38–42%, respectively [49,50]. Therefore, the elastic recovery ratios of ZnSe are very close to those of zinc-blende AlN and GaN.

### 3.3. The Correlation of Structural Evolution and Mechanical Responses

**Figure 5:**
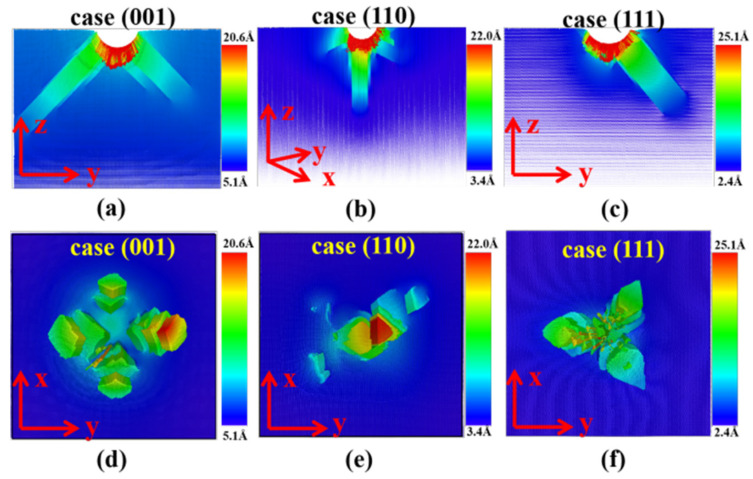
(**a**–**c**) The atomic displacement vectors in a slice (thickness of 1 nm) that passing through the center of the spherical indenter at h = 5.0 nm. (**d**–**f**) The bottom views of the atomic displacement vectors in a slice (1 nm thick) at the position of 8.5 nm below the indented planes.

The mechanical responses during the indenting process should be closely related with the microstructure evolutions of the indented substrates. To understand the fundamental mechanisms of the indenting responses, the displacement vectors of the particles in the indented substrates were systematically analyzed, as shown in Figure 5. The displacement vectors of particles were calculated based on two snapshots of the system: the initial configuration and the final configuration when the indentation depth reached 5 nm during the indenting process. The displacement vector of a particle was computed by subtracting the initial position from its final position, and can be represented by an arrow. Therefore, the directions of the atomic displacements can be represented by the directions of the arrows, and the magnitudes of the displacements can be represented by the length of the arrows. Figure 5a–c show the atomic displacement vectors in a slice (thickness 1 nm) that passing through the center of the spherical indenter of the (001), (110), and (111) samples. Figure 5d–f show the bottom views of the atomic displacement vectors in a slice (1 nm thick) at the position of 8.5 nm below the indented (001), (110), and (111) planes. The figures in Figure 5 are actually composed of many arrows. In addition, these displacement vectors (arrows) are color coded according to their magnitudes. To see the arrows more clearly, we put the enlarged version of these figures in the supplemental materials, as shown in Figure S2. As can be seen in Figure 5, the atomic displacements of the indented samples show the following three characteristics. First, due to the compression effect of the indenter, atoms of the samples have a certain degree of downward displacement, and the atoms with larger displacements are always distributed in the contact area below the indenter. In addition, atoms around the indenter tend to move along the radial direction of the indenter. Second, as shown in Figure 5d–f, although the atoms at the position of 3.5 nm below the indentation have no direct contact with the indenter, the displacements of some atoms can reach about 2 nm. Third, the displacement directions of these atoms show specific directions due to the different crystal planes. As illustrated in the figure, for the atoms at 3.5 nm below the indented (001), (110), and (111) crystal planes, large atomic displacements can be found in 4, 3, and 3 directions, respectively, and has obvious symmetry.

**Figure 6:**
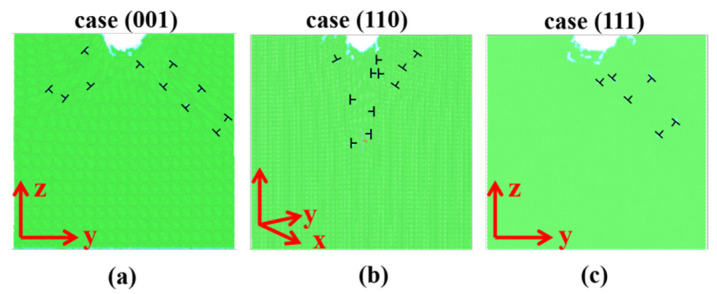
Atomic arrangement and dislocation distributions under the indented planes. (**a**) under the indented (001) plane, (**b**) under the indented (110) plane, and (**c**) under the indented (111) plane.

It is found that for all the cases, although the indenting responses are different, the plastic deformations mechanisms are demonstrated to be the same. That is, the plastic deformations under all the indented crystalline planes are dominated by the dislocation nucleation and propagations. Figure 6 shows the atomic arrangement of a slice (thickness of 1 nm) that passing through the center of the spherical indenter of the (001), (110), and (111) cases. As can be seen in the figure, under the indentations, dislocations are found appeared under all the indented samples. These dislocations are found not only appeared around the indentations, but also propagated far away from the indenter. In addition, the directions that the dislocation propagated in the indented samples are basically consistent with the direction of the atomic displacement vector illustrated in Figure 5. Therefore, we speculate that the large and regular displacements of atoms in the indented samples are closely related to the dislocation activities.

**Figure 7:**
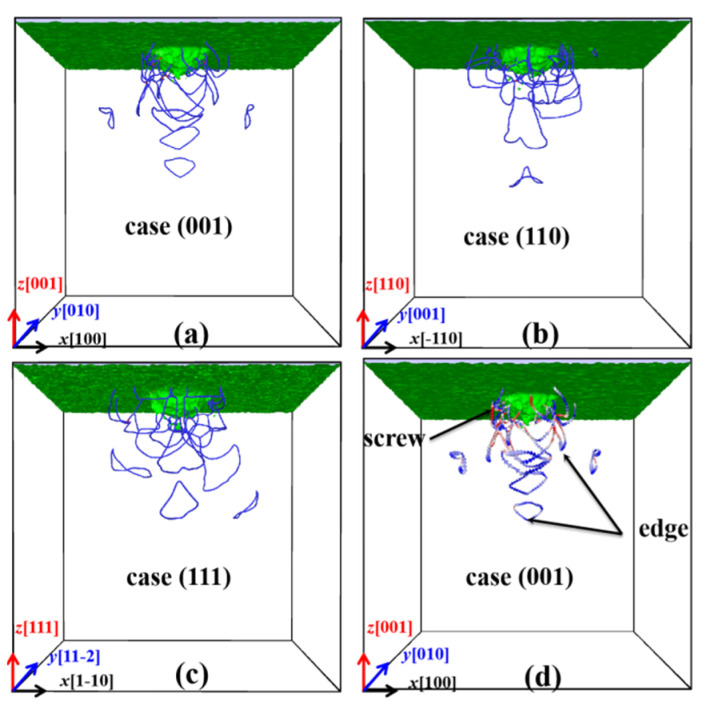
Dislocations of the indented ZnSe nano films with maximum indentation depth of 5.0 nm. Blue lines in (**a**–**c**) represent the 1/2<110> type dislocations. Blue, red, and white arrows in (**d**) represent the edge, screw, and mixed dislocations.

The dislocations of the indented ZnSe nano films with indentation depth of 5 nm are analyzed through DXA method. As shown in Figure 7a–c, the dislocations under the indented planes are represented by the blue lines, which are identified to be the 1/2<110> type dislocations. It can be seen from Figure 7a–c that the dislocations in all cases of this work are mainly the 1/2<110> type dislocations, but their distributions are quite different. Aimed to illustrate the characteristics of the dislocations, we further analyzed in Figure 7d the geometric structure of the extracted shear loop by DXA, where the edge, screw and the mixed dislocations are represented by blue, red, and white arrows. It is found that most of the dislocation lines consist of edge, screw, and mixed dislocations. In addition, most of the screw components are connected with the surface or the indentation, while the edge components are located at the front end of the dislocations.

**Figure 8:**
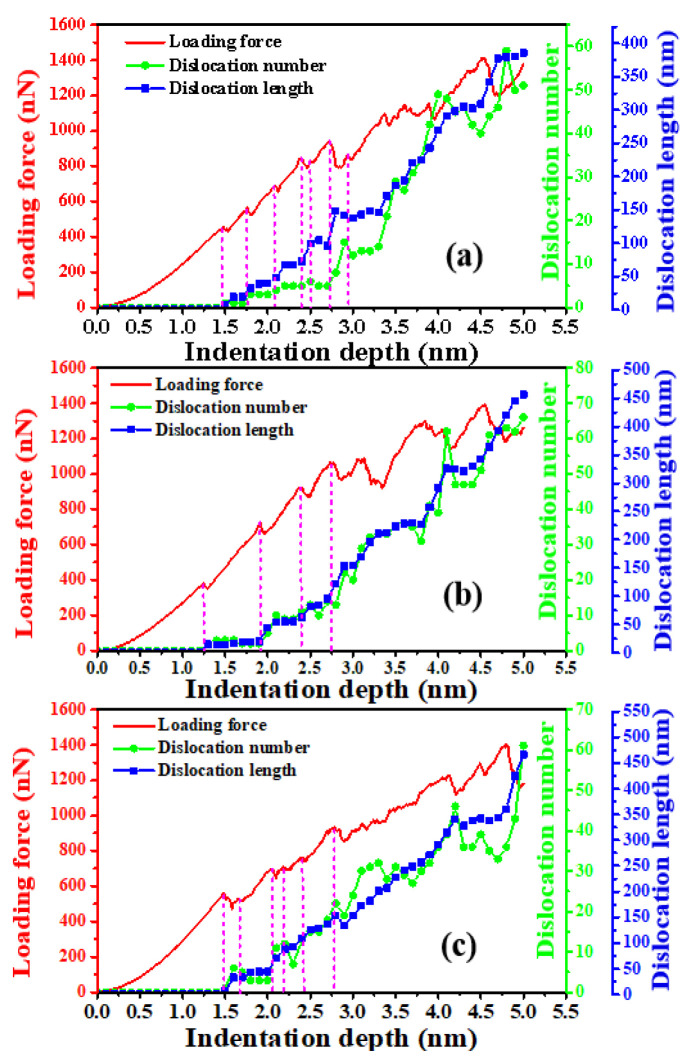
The dislocation length, dislocation number, and the loading force as a function of indentation depth: (**a**) (001) plane, (**b**) (110) plane and (**c**) (111) plane.

The correlation of the dislocation length, dislocation numbers, as well as the loading force during the penetrating processes have been evaluated, as shown in Figure 8. Generally, the evolution of the dislocation numbers and dislocation length of the indented substrates during the loading process can be mainly divided into four stages. In the first stage, the loading force increases with the indentation depth, but no dislocations can be detected in the indented samples. In this stage, the area contacted by the indenter will deviate from the original plane of the substrates by more than 1 nm, but the deviation range is still within the elastic deformation range of the material. If the indenter is withdrawn at this stage, the corresponding area will be completely restored. Thus, the first stage is the elastic deformation stage. In the second stage, dislocations begin to appear and present the characteristics of stepped increase with the increase of indentation depth. As shown in the figure, in this stage, when the indentation depth is increased to a critical value, the dislocations appear, indicating the occurrence of the plastic deformations. Interestingly, the number and length of dislocations do not increase slowly with the increase of indentation depth. Instead, the number and length of dislocations increase rapidly to a certain amount. After that, they remain unchanged in a certain indentation depth range. Until another critical point is reached, the values of dislocation number and dislocation length would increase rapidly again to another amount, and remain almost constant in an indentation depth range. In this stage, the increase of dislocation number and length is directly related to the sudden decrease of loading force at certain indentation depth. In addition, for different indented crystalline planes, the indentation depth range that the second stage covered is different. For example, in the indentation depth range from 1.48 nm to 3.35 nm, the dislocation behavior under the indented (001) plane conforms to the characteristics of the second stage. For the indented (110) and (111) planes, the variation laws of dislocation numbers and length in the indentation depth range from 1.25 nm to 2.73 nm and from 1.49 nm to 2.05 nm conform to the characteristics of the second stage. In the third stage, with the increase of indentation depth, the characteristic of step increase for the dislocation number and length gradually disappears. Instead, they increase almost linearly with the increase of indentation depth. We take the indented (001) plane as an example. In the indentation depth range from 3.59 nm to 3.81 nm, the variation of the dislocation number and length with the indentation depth conforms to the characteristics of the third stage. In the fourth stage, with the increase of indentation depth, the dislocation length continues to increase, but the number of dislocations fluctuates with the increase of indentation depth. During the indenting process of ZnSe, the produced dislocations will react and form prismatic loops. Thus, we speculate that two main factors may give rise to the fluctuation of the dislocation number: The first one is the formations and releases of prismatic loops. The second one is the interactions between the dislocations, which lead to the annihilation and contraction of dislocations.

### 3.4. Dislocation Activities of ZnSe Nano Films under Indentations

**Figure 9:**
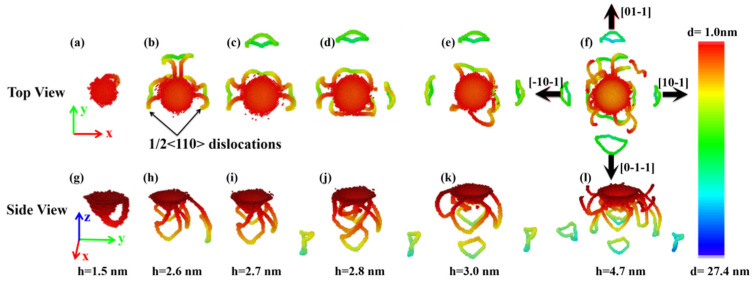
The microstructure evolution under the indented (001) plane of ZnSe nano film at different indentation depth. Atoms are colored with z-value. (**a**), (**b**), (**c**), (**d**), (**e**), and (**f**) are the top views of the structures at the indentation depth of 1.5 nm, 2.6 nm, 2.7 nm, 2.8 nm, 3.0 nm, and 4.7 nm, respectively. (**g**), (**h**), (**i**), (**j**), (**k**), and (**l**) are the side views of the structures at the indentation depth of 1.5 nm, 2.6 nm, 2.7 nm, 2.8 nm, 3.0 nm, and 4.7 nm, respectively.

**Figure 10:**
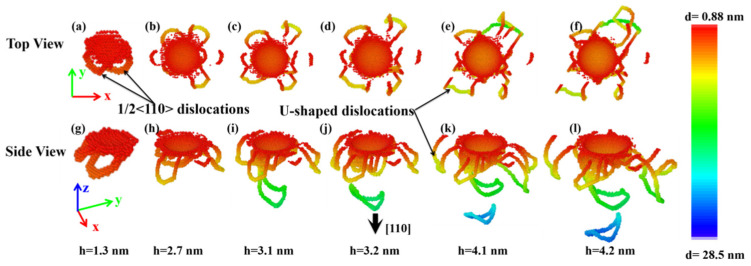
The microstructure evolution under the indented (110) plane of ZnSe nano film at different indentation depth. Atoms are colored with z-value. (**a**), (**b**), (**c**), (**d**), (**e**), and (**f**) are the top views of the structures at the indentation depth of 1.3 nm, 2.7 nm, 3.1 nm, 3.2 nm, 4.1 nm, and 4.2 nm, respectively. (**g**), (**h**), (**i**), (**j**), (**k**), and (**l**) are the side views of the structures at the indentation depth of 1.3 nm, 2.7 nm, 3.1 nm, 3.2 nm, 4.1 nm, and 4.2 nm, respectively.

**Figure 11:**
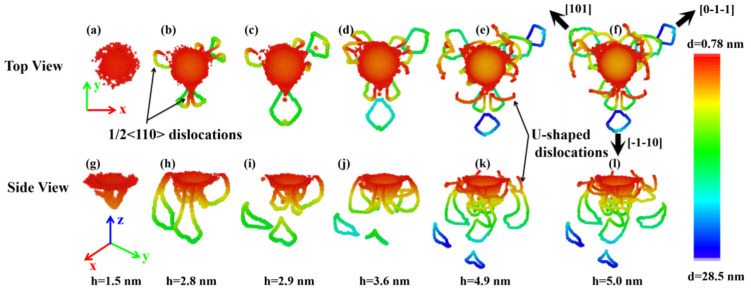
The microstructure evolution under the indented (111) plane of ZnSe nano film at different indentation depth. Atoms are colored with z-value. (**a**), (**b**), (**c**), (**d**), (**e**), and (**f**) are the top views of the structures at the indentation depth of 1.5 nm, 2.8 nm, 2.9 nm, 3.6 nm, 4.9 nm, and 5.0 nm, respectively. (**g**), (**h**), (**i**), (**j**), (**k**), and (**l**) are the side views of the structures at the indentation depth of 1.5 nm, 2.8 nm, 2.9 nm, 3.6 nm, 4.9 nm, and 5.0 nm, respectively.

To shed light on the nucleation and propagation of dislocations, we analyzed the propagation details of dislocations under the indented (001), (110), and (111) planes of ZnSe nano films, as shown in Figure 9, Figure 10 and Figure 11. The microstructures of the indented samples at different indentation depth are analyzed through the IDS method [39], and the top view and side view of the indented samples are illustrated. In these figures, the atoms with zinc blende lattice have been removed for clarity, and the atoms are color coded according to distances from them to the top surface of the indented substrates (d shown in these figures). The dislocation lines under the indented (001), (110), and (111) planes of ZnSe nano films are also obtained through the DXA method [40] and are shown in the Supplemental Materials in Figures S3–S5, respectively.

As can be seen in Figure 9a,g, for the indented (001) plane of ZnSe, dislocations with slip system of {111}<110> nucleated after the indenter penetrated into the depth of about 1.5 nm. Such indentation depth agrees well with that of the first load drop (pop-in event) in the load-displacement curve shown in Figure 3, indicating that the pop-in event is indeed correlated with the nucleation of dislocations. Thus, at the indentation depth of about 1.5 nm, the area contacted with the indenter has experienced unambiguous transitions from reversible elastic to irreversible plastic deformations. As the indenting process continues, more and more dislocations of {111}<110> slip systems nucleated around the indentation. As shown in Figure 9b,h, at the indentation depth of 2.6 nm, dislocations are found closely connected with the indentation, and symmetrically distributed in [10-1], [-10-1], and [01-1] directions. When the indenter was further pressed down to 3.0 nm, dislocations are also found nucleated in [0-1-1] direction. Therefore, for the indented (001) plane of ZnSe nano film, dislocations nucleate and propagate mainly in four directions. After nucleation and evolution, dislocations would react and form prismatic loops in their respective propagation directions. Subsequently, they are detached and emitted into the substrates along [10-1], [-10-1], [01-1], and [0-1-1] directions. As shown in Figure 9f,l, the emitted prismatic loops around the indentation show a typical fourfold symmetric distribution. It is worth noting that with the continuous pressing of the indenter, dislocations will continue to nucleate and grow, and prismatic loops will continuously emit along these four directions, as shown in Figure 7a.

For the indented (110) sample, its primary mechanism of plastic deformation is still the nucleation and propagation of dislocations. As shown in Figure 10a,g, at the initial stage of plastic deformation, a small amount of dislocations begin to nucleate, and distribute below and on the side of the indentation. Although the dislocation nucleates and propagates only in a small area around the indentation, it is enough to effect the force of the indenter, resulting in the first load drop of the corresponding p-h curve, as shown in Figure 3. These dislocations are identified to be the 1/2<110> type and are made up of screw and edge components. As the indenter continues to press down, these dislocations would propagate away from the indentation. Moreover, new dislocations would nucleate around the indentation. Generally, the dislocation behaviors under the indented (110) crystalline plane of ZnSe nano film can be divided into two categories. For the first category, the propagation direction of these dislocations is along the loading direction of the indenter. That is the <110> direction. With the increase of indentation depth, these dislocations will not only propagate along the pressing direction of the indenter, but also release prismatic loops right below the indentation, as shown in Figure 10j. The emitted prismatic loops will propagate deep into the sample after they detached away from the contacted dislocations. For the other category, dislocations are found multiplied and glide along the direction parallel to the indented (110) plane. When these dislocations nucleate and grow to a certain extent, they will also detach away from the area around the indentation. Different from the first propagation mode, the dislocations of this category will form U-shaped dislocations rather than complete dislocation loops and propagate far away from the indenting area. In the meantime, the emitted U-shaped dislocations are always in contact with the indented surface of the sample, as shown in Figure 10j–l.

Figure 11 displays the evolution of defects induced by indentation on the (111) plane of ZnSe nano film at different depth. It is found that the plastic deformation of the indented (111) plane of ZnSe can also be attributed to the nucleation and propagation of 1/2<110> type dislocations. When the indenter is pressed into the (111) plane at a depth of about 1.5 nm, the plastic deformation of the indented area are initiated, and a few dislocations are found nucleated right below the indented area, as shown in Figure 11a,g. With the increase of indentation depth, dislocations would nucleate and grow near the indented area. At the same time, some dislocations are also found propagate away from the indentation area. Due to different crystal orientations, the dislocation evolution process under the indented (111) plane of ZnSe is different from those of (001) plane and (110) plane. Firstly, the propagation directions of dislocations are different from those of (001) plane and (110) plane. As can be seen in the top view of the dislocation distributions in Figure 11, the dislocation propagation below the indented (111) plane shows an obvious triple symmetrical distribution. Comparing with the results shown in Figure 5f, we can see that the dislocation propagation directions are consistent with the displacement vector distribution of the atoms below the indentation. Second, the modes of dislocation propagation are different. With the increase of indentation depth, the dislocations below the indented (111) plane would emit prismatic loops, which would propagate obliquely downward. At the same time, the dislocations near the indentation area will form U-shaped dislocations, which would propagate in the direction parallel to the surface, as shown in Figure 11f,l. The dislocation propagation mode of (111) plane differing from those of (001) and (110) planes is as follows. For (001) case, there is no U-shaped dislocations propagating along the indented plane. For the (110) case, there are both U-shaped dislocations and the emitted prismatic loops below the indented plane, but their propagation directions are completely inconsistent. In comparison, for the (111) case, the directions of the U-shaped dislocation and the emitted prismatic loops in the XY dimensions are basically the same, as shown in Figure 11f.

### 3.5. Formation Mechanisms of the Prismatic Loops

**Figure 12:**
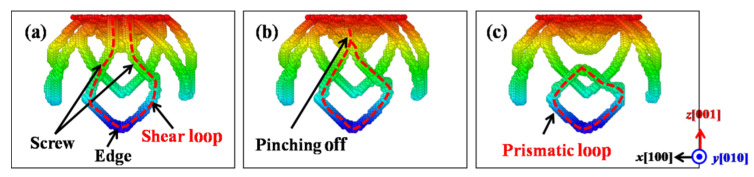
The formation process of prismatic loops based on the “lasso”-like mechanism observed under the indented (001) crystalline plane of ZnSe. Color scale from red to blue indicating depth from the indented plane. The dash line depicts the related shear loop for the formation of prismatic loops. (**a**) Screw and edge components in the shear loops. (**b**) Two screw components start to merge and prismatic loop has formed. (**c**) The formed prismatic loop has pinched off from the indentation region.

As mentioned above, the prismatic loops are found emitted under all the indented crystalline plane and play an important role in the plastic deformations of ZnSe nano films. Therefore, the formation mechanisms of these prismatic loops will be explored. Figure 12 presents the formation process of prismatic loops under the indented (001) crystalline plane of ZnSe. As can be seen in Figure 12a, the shear loops are made up of screw and edge component. The screw components of the shear loop have opposite signs, and are directly connected with the indenting area. In the initial stage of the formation process of the prismatic loops, the screw components are almost perpendicular to the indentation plane, while the edge components are almost parallel with the indented plane. As the indenting process continued, the edge component of the dislocation will gradually glide away from the indentation region due to the effect of the indentation stress field, while the screw components start to attract each other and cross slip due to the dragging force from the edge dislocations. Therefore, these two screw components start to merge, and the prototype of the prismatic loop has gradually formed, as shown in Figure 12b. When the indenter continues to press down, the merged prismatic loop will pinch off from the indentation region, and glide away driven by the indentation induced stress. From the formation process of the prismatic loops under the indented (001) plane of ZnSe, we find that it belongs to the typical “lasso”-like formation mechanism, which can be found under the indentations of bcc metals like Ta [51] and Fe [52,53], as well as other materials such as AlN [25], GaN [25,50], 3C-SiC [54].

**Figure 13:**
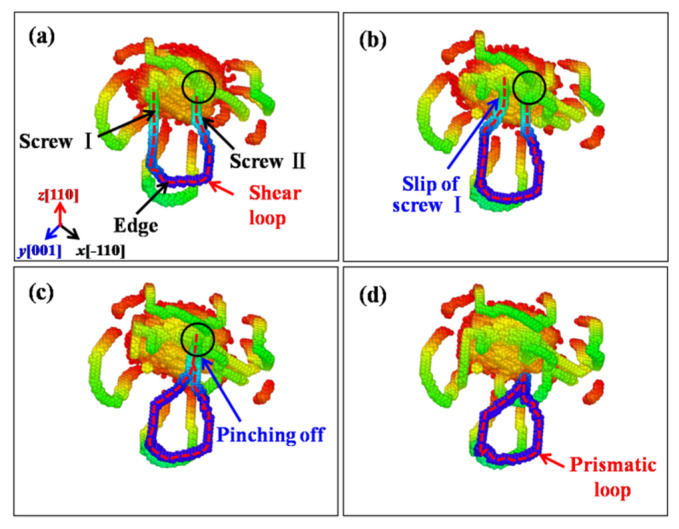
Formation of prismatic loop based on extended “lasso”-like mechanism for indentation on (110) plane, with colors scale from red to blue indicating depth from sample surface. The dash line depicts the related shear loop for formation of the prismatic loop. The black circles represent the position where two screws intersect with each other. (**a**) Screw and edge components in the shear loop. (**b**) The glide of screw component in the shear loop. (**c**) Two screw components encounter each other, and a prismatic loop has formed. (**d**) The formed prismatic loop has pinched off from the indentation region.

In Figure 13, we present the formation process of prismatic loops under the indented (110) plane of ZnSe, which can be depicted by the extended “lasso”-like mechanisms. It is slightly different from the “lasso”-like mechanisms in the (001) case. As shown in Figure 13a,b, for the two screw components of a shear loop, the screw II component remains almost stationary, while the screw component I can glide on another shear loop. Driven by the stress filed under the indentation, these two screw components will encounter each other, followed by pinching off and gliding away, resulting in the emitting of the prismatic loop, as shown in Figure 13c,d.

**Figure 14:**
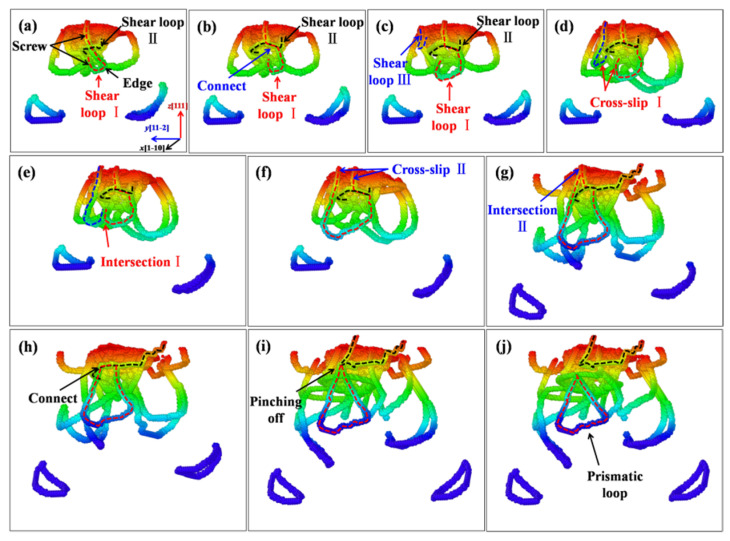
Formation of prismatic loops based on “nested-loops” mechanism for indentation on (111) plane, with colors scale from red to blue indicating depth from sample surface. The dash lines depict the related shear loops for formation of the prismatic loop. (**a**–**c**) The nucleated shear loop I, II, and III, respectively. (**d**) Screw components of shear loop I and III start to cross-slip. (**e**) Shear loop I and III glide to the same position. (**f**) Cross slip of shear loop II and III. (**g**) Intersection of shear loop II and III. (**h**) Merging of shear loop II and III. (**i**) The merged dislocation loop starts to break away from the indented area. (**j**) The emitted prismatic loop.

The “lasso”-like formation mechanism of the prismatic loops is also found under the indented (111) crystal plane of ZnSe. In addition, another formation mechanism is observed, which is different from those of the (001) and (110) cases. For the “lasso”-like and extend “lasso”-like mechanisms, the two screw components from the same shear loop will intersect with each other, and produce a whole prismatic loop. In comparison, under the indented (111) plane of ZnSe, some of the emitted prismatic loops are formed by connecting multiple shear loops. Figure 14 shows the detailed process of an emitted prismatic loop, which is formed by the intersection and merging of three shear loops. As shown in Figure 14a–c, three adjacent shear loops I, II, and III are nucleated one after another around the indentation. Driven by the stress field induced by the indenting process, the screw component of shear loop I firstly connected with the edge component of shear loop Ⅱ, as shown in Figure 14b. With the proceeding of indentation, shear loop III nucleated and propagated gradually, and began to participate in the formation of the prismatic loops. As can be seen in Figure 14c–e, while the shear loop III is growing up, it gradually propagates close to the shear loop I. When they meet with each other, the screw components of shear loop I and III start to cross-slip (Figure 14d) and glide to the same position, resulting in the merging of these two shear loops (Figure 14e,f). After that, the screw components of the shear loop II and III would experience the similar cross-slip (Figure 14f) and intersection (Figure 14g) processes. During the approaching process of shear loops II and III, the screw component of shear loop I connected with Loop II would be dragged towards shear loop III, resulting in the merging of these two shear loops, as shown in Figure 14f–h. So far, the prototype of prismatic loop composed of three shear loops has been basically formed. When continuing to increase the indentation depth, the merged dislocation loop will break away from the original dislocation structure and be emitted deep into the sample, as shown in Figure 14i,j. Previously in the literature, when Xiang et al. investigated the indentation behaviors of AlN and GaN, they also found a similar result [25]. That is, the multiple shear loops adjacent to each other can form a nested structure by cross-slip, and then the prismatic loop formed by the interact of the screw components of two shear loops. Therefore, the formation mechanisms of the emitted prismatic loops under the indented (111) crystalline plane of ZnSe can be attributed to both the”lasso”-like formation mechanism and the so called “nested-loops” mechanism.

## 4. Conclusions

In summary, through molecular dynamics simulations, we have investigated the indentations on the (001), (110), and (111) planes of ZnSe nano films and analyzed the corresponding plastic deformation mechanisms. The mechanical responses of ZnSe during the indenting process have been evaluated by the loading force versus indentation depth curves. We find that the elastic modulus of the (111) plane is the highest, while that of the (001) plane is the lowest. The indentation induced incipient plasticity occurs first on the (110) plane, then the (001) plane and, finally, the (111) plane. Due to the elastic recovery of the indented planes during the retracting process of the indenter, the final depths of the indentations are different. We find that the elastic recovery ratios of the indented planes of ZnSe are in the range of 37–47%. To analyze the correlation of structural evolution and mechanical responses, the atomic displacement vectors, atomic arrangements, and the dislocations of the indented samples are analyzed. We have revealed that the plastic deformations of the indented ZnSe nano films are dominated by the nucleation and propagation of 1/2<110> type dislocations, and the symmetrically distributed prismatic loops emitted during the indenting process are closely related with the mechanical properties. By studying the evolutions of microstructures, the formation process of the dislocations, as well as the formation mechanisms of the emitted prismatic loops under the indented crystalline planes, are discussed. The results presented in this work not only provide an answer for the questions about indentation responses of ZnSe on (001), (110), and (111) planes, but also offer insight into the plastic deformation mechanisms. This work provides reference data to improve the design and ultimately performance of the related ZnSe based devices.

## Figures and Tables

**Table 1:** The indented planes, dimensions, and numbers of atoms for the simulated ZnSe nano film models in this work.

Indented Plane	Dimensions	Orientation	L (nm)	Total Atoms
(001)	x	[100]	57.73	8,000,000
	y	[010]	57.73	
	z	[001]	57.73	
(110)	x	[−110]	57.97	8,065,600
	y	[001]	57.73	
	z	[110]	57.97	
(111)	x	[1-10]	57.97	8,104,224
	y	[11-2]	57.98	
	z	[111]	57.99	

**Table 2:** Properties of ZnSe calculated by the selected interatomic potential in this work, compared with the reported data from experiments and first principles calculations.

Properties	This Work	Experiment	First Principles
Lattice constant a (Å)	5.668	5.667 [42]	5.605 [43], 5.545 [44], 5.624 [45]
Elastic constant C_11_ (GPa)	92.4	88.8 [42]	96.12 [43], 110.34 [44], 95.9 [45]
Elastic constant C_12_ (GPa)	47.3	52.7 [42]	57.36 [43], 63.02 [44], 53.6 [45]
Elastic constant C_44_ (GPa)	30.1	41.4 [42]	44.75 [43], 49.06 [44], 48.9 [45]
Bulk modulus (GPa)	62.3	62.5 [42]	70.29 [43], 78.79 [44], 71.82 [45]
Shear Modulus (GPa)	30.2	29.1 [42]	31.98 [43], 36.61 [44], 24.3 [45]
Youngs modulus (GPa)	71.0	74.6 [42]	83.25 [43], 95.10 [44], 63.8 [45]
Poisson’s ratio	0.33		0.3014 [43], 0.30 [44], 0.31 [45]

## Data Availability

The data used to support the findings of this study are available from the corresponding author upon request.

## Supplementary Materials

The following are available online at https://www.mdpi.com/article/10.3390/nano11113014/s1, Figure S1: Generalized stacking fault energy as a function of the relative shear displacement. (a) (111)<1-10>; (b) (111)<11-2>; Figure S2. The atomic displacement vectors in a slice (thickness of 1 nm) that passing through the center of the spherical indenter at h = 5.0 nm. (a) Case (001); (b) Case (110); (c) Case (111); Figure S3. The evolution process of the dislocations under the indented (001) plane of ZnSe nano film: (a) h = 1.5 nm, (b) h = 2.6 nm, (c) h = 2.7 nm, (d) h = 2.8 nm, (e) h = 3.0 nm, (f) h = 4.0 nm, (g) h = 4.5 nm and (h) h = 4.7 nm; Figure S4. The evolution process of the dislocations under the indented (110) plane of ZnSe nano film: (a) h = 1.3 nm, (b) h = 2.7 nm, (c) h = 3.1 nm, (d) h = 3.2 nm, (e) h = 4.1 nm and (f) h = 4.2 nm; Figure S5. The evolution process of the dislocations under the indented (111) plane of ZnSe nano film: (a) h = 1.5 nm, (b) h = 2.8 nm, (c) h = 2.9 nm, (d) h = 3.6 nm, (e) h = 4.9 nm and (f) h = 5.0 nm.
