# Supplementary material for: Orientation Dependent Mechanical Responses and Plastic Deformation Mechanisms of ZnSe Nano Films under Nanoindentation
Source: Nanomaterials (Basel). 2021 Nov 10;11(11):3014. doi: 10.3390/nano11113014 (PMC8619347; doi:10.3390/nano11113014)
Supplement: Supplementary file 1 [file nanomaterials-11-03014-s001.zip › nanomaterials-1456494-supplementary.pdf]

nanomaterials

Supplementary Materials

Orientation Dependent Mechanical Responses and Plastic Deformation Mechanisms of ZnSe Nano Films under Nanoindentation

Chao Xu [1,2,3], Futi Liu [1,2,*], Chunmei Liu [3,*], Pei Wang [4] and Huaping Liu [5]

1 Faculty of Science, Yibin University, Yibin 644007, China; chaoxu@wust.edu.cn
2 Key Laboratory of Computational Physics, Yibin University, Yibin 644007, China
3 College of Science, Wuhan University of Science and Technology, Wuhan 430081, China
4 Academy for Advanced Interdisciplinary Studies, Department of Physics, Southern University of Science and Technology (SUSTech), Shenzhen 518055, China; pwsustech@outlook.com
5 School of Aerospace Engineering, Huazhong University of Science and Technology, Wuhan 430074, China; huaping_liu@163.com
* Correspondence: liufuti001@163.com (F.L.); liuchunmei@wust.edu.cn (C.L.)

Citation: Xu, C.; Liu, F.; Liu, C.; Wang, P.; Liu, H. Orientation Dependent Mechanical Responses and Plastic Deformation Mechanisms of ZnSe Nano Films under Nanoindentation. *Nanomaterials* **2021**, *11*, 3014. https://doi.org/10.3390/10.3390/nano11113014

Academic Editors: Yang-Tse Cheng and Jordi Sort

Received: 25 October 2021
Accepted: 5 November 2021
Published: 10 November 2021

Publisher's Note: MDPI stays neutral with regard to jurisdictional claims in published maps and institutional affiliations.

Copyright: © 2021 by the authors. Licensee MDPI, Basel, Switzerland. This article is an open access article distributed under the terms and conditions of the Creative Commons Attribution (CC BY) license (http://creativecommons.org/licenses/by/4.0/).

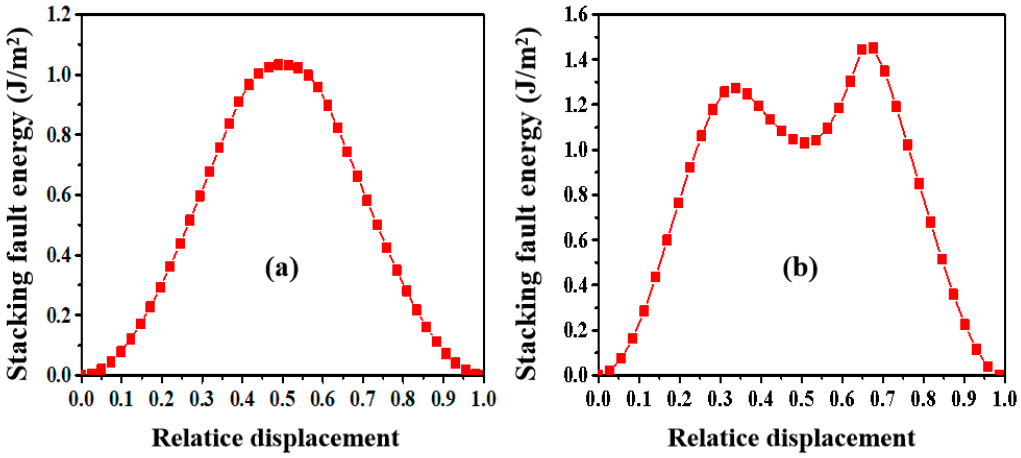

Figure S1. Generalized stacking fault energy as a function of the relative shear displacement. **(a)** (111)<1-10>; **(b)** (111)<11-2>.

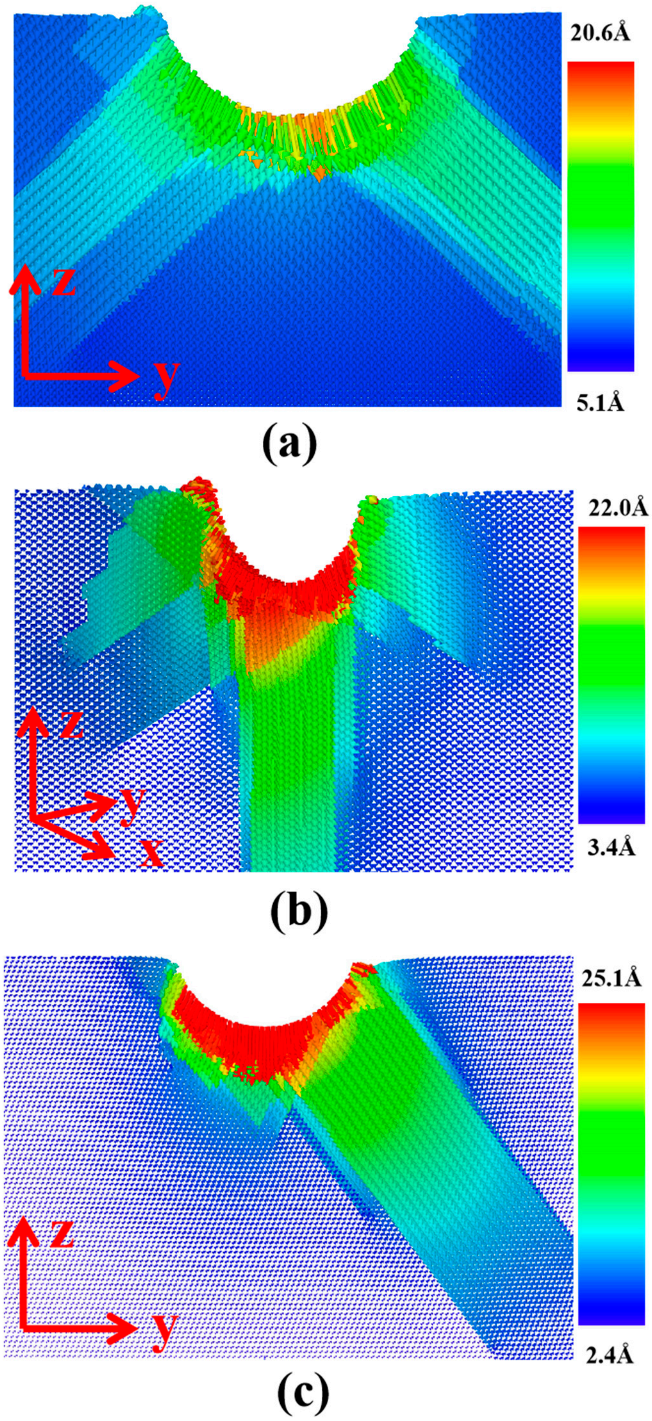

Figure S2. The atomic displacement vectors in a slice (thickness of 1 nm) that passing through the center of the spherical indenter at h = 5.0 nm. **(a)** Case (001); **(b)** Case (110); **(c)** Case (111).

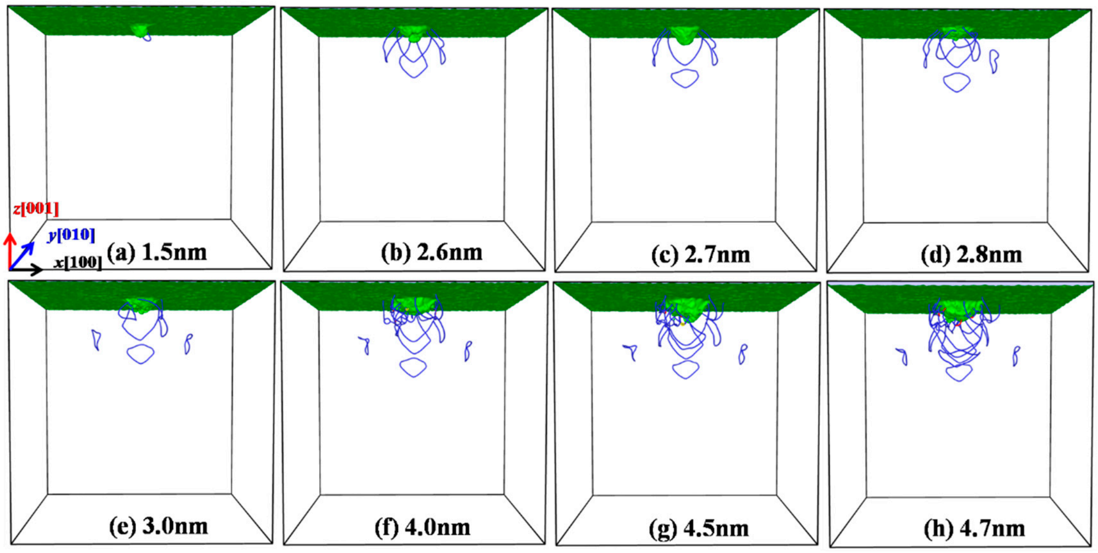

Figure S3. The evolution process of the dislocations under the indented (001) plane of ZnSe nano film: **(a)** h = 1.5 nm, **(b)** h = 2.6 nm, **(c)** h = 2.7 nm, **(d)** h = 2.8 nm, **(e)** h = 3.0nm, **(f)** h = 4.0 nm, **(g)** h = 4.5 nm and **(h)** h = 4.7 nm.

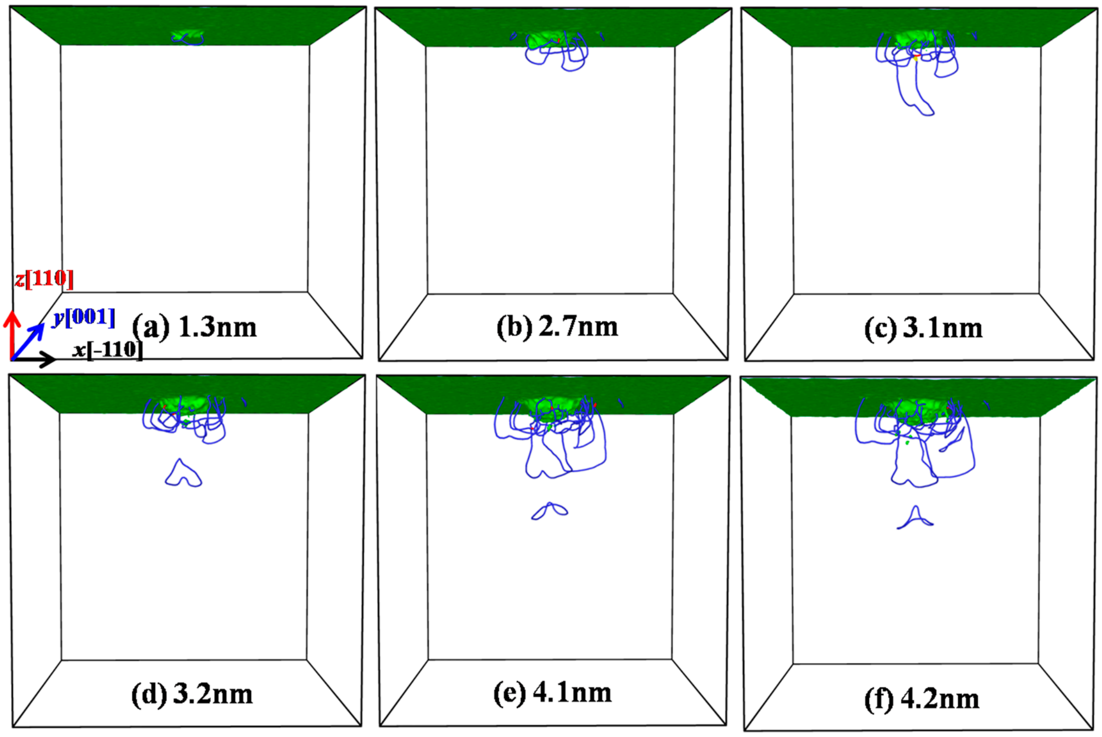

Figure S4. The evolution process of the dislocations under the indented (110) plane of ZnSe nano film: **(a)** h = 1.3 nm, **(b)** h = 2.7 nm, **(c)** h = 3.1 nm, **(d)** h = 3.2 nm, **(e)** h = 4.1nm and **(f)** h = 4.2 nm.

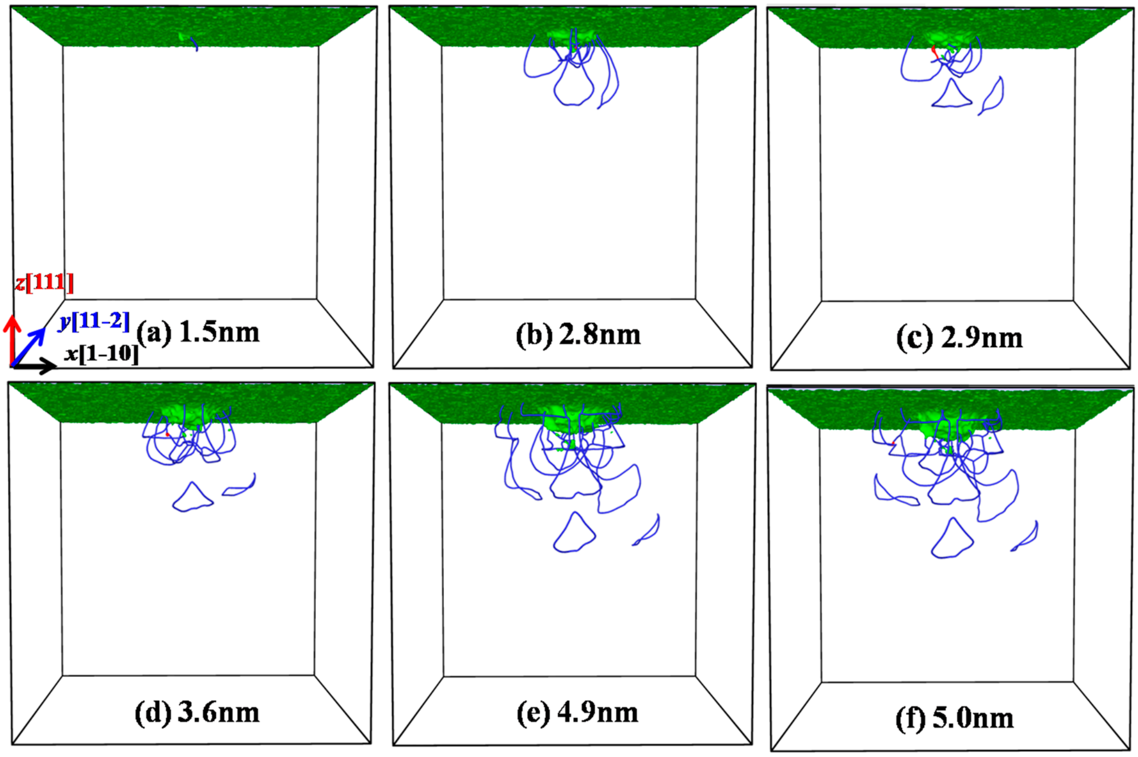

Figure S5. The evolution process of the dislocations under the indented (111) plane of ZnSe nano film: **(a)** h = 1.5 nm, **(b)** h = 2.8 nm, **(c)** h = 2.9 nm, **(d)** h = 3.6 nm, **(e)** h = 4.9 nm and **(f)** h = 5.0 nm.
